# N-Terminal Extension and C-Terminal Domains Are Required for ABCB6/HMT-1 Protein Interactions, Function in Cadmium Detoxification, and Localization to the Endosomal-Recycling System in *Caenorhabditis elegans*

**DOI:** 10.3389/fphys.2018.00885

**Published:** 2018-07-30

**Authors:** Sungjin Kim, Anuj K. Sharma, Olena K. Vatamaniuk

**Affiliations:** ^1^Section of Soil and Crop Sciences, School of Integrative Plant Science, Cornell University, Ithaca, NY, United States; ^2^Section of Plant Biology, School of Integrative Plant Science, Cornell University, Ithaca, NY, United States

**Keywords:** heavy metals, cadmium, HMT-1, ABCB6, ABC transporters, recycling endosomes, *C. elegans*

## Abstract

The chronic exposure of humans to toxic metals such as cadmium from food and air causes dysfunction of vital organs, neurodegenerative conditions, and cancer. In this regard, members of the ABCB sub-family of the ATP-binding cassette (ABC) transporter superfamily, ABCB6/HMT-1, are acutely required for the detoxification of heavy metals and are present in genomes of many organisms including the nematode worm, *Caenorhabditis elegans* and humans. We showed previously that *C. elegans* ABCB6/HMT-1 detoxifies cadmium, copper, and arsenic, and is expressed in liver-like cells, the coelomocytes, head neurons and intestinal cells, which are the cell types that are affected by heavy metal poisoning in humans. The subcellular localization of ABCB6/HMT-1 proteins is unclear. ABCB6/HMT-1 proteins have a distinguishing topology: in addition to one transmembrane domain and one nucleotide-binding domain, they possess a hydrophobic N-terminal extension (NTE) domain encompassing five to six transmembrane spans. The role of the NTE domain in the function of ABCB6/HMT-1 in the native organism remains to be investigated. We used a versatile, multicellular model system, *C. elegans*, to establish the subcellular localization of ABCB6/HMT-1 and refine its structure-function studies in the native organism. We show that ABCB6/HMT-1 localizes mainly to the apical recycling endosomes and, in part, to early and late endosomes of intestinal cells. We also show that ABCB6/HMT-1 lacking the NTE domain is mistargeted to the plasma membrane and is unable to confer cadmium resistance. Although the NTE domain is essential for ABCB6/HMT-1 interaction with itself, the absence of NTE does not fully prevent this interaction. As a result, ABCB6/HMT-1 lacking the NTE domain, and expressed in wild-type worms or co-expressed with the full-length polypeptide, inactivates and mistargets the full-length ABCB6/HMT-1. We also show that the 43 amino acid residue stretch at the COOH-terminus is required for the ABCB6/HMT-1 interaction with itself and cadmium detoxification function. These results suggest that both NTE and COOH-terminus must be present to allow the protein to interact with itself and confer cadmium resistance. Considering that ABCB6/HMT-1 proteins are highly conserved, this study advances our understanding of how these proteins function in cadmium resistance in different species. Furthermore, these studies uncover the role of the endosomal-recycling system in cadmium detoxification.

## Introduction

Cadmium (Cd) is a highly toxic transition metal that poses a threat to human health and environment (Jarup, 2003; Horiguchi et al., 2010). It is now acknowledged that the chronic exposure to Cd is associated with the dysfunction of vital organs and can cause cancer (Joseph, 2009; Horiguchi et al., 2010). At the cellular level, Cd toxicity results from the displacement of the endogenous co-factors from their cellular binding sites, thiol capping of essential proteins, inhibition of DNA repair processes, and interference with reactive oxygen species detoxifying system (Stadtman, 1990;Waisberg et al., 2003; Valko et al., 2005). In addition to Cd, other heavy metals and metalloids [e.g., cadmium (Cd), mercury (Hg), lead (Pb), and arsenic (As)] are of considerable biological and environmental concern (Waalkes et al., 1992; Waalkes, 2003; Baron et al., 2006).

Members of the highly conserved family of ATP-binding cassette (ABC) transporters are recognized for their contribution to heavy metal detoxification in different species (Ortiz et al., 1992; Broeks et al., 1996; Jalil et al., 2008; Preveral et al., 2009; Sooksa-Nguan et al., 2009; Kim et al., 2010; Song et al., 2014). For example, plasma membrane-localized ABCC1/MRP1 and ABCC2/MRP2 of humans confer cellular efflux of arsenic-glutathione (GSH) conjugates (Leslie et al., 2004; Leslie, 2012). The HsABCC1 and HsABCC2 counterpart in yeast *Saccharomyces cerevisiae*, Ycf1p (**Y**east **C**admium **f**actor 1), detoxifies heavy metals including Cd and As by sequestering metal-GS complexes into a lysosomal-like compartment, the vacuole, and confers heavy metal tolerance in heterologous systems (Li et al., 1996, 1997; Bhuiyan et al., 2011). ABCB6-like proteins [alias HMT-1 (**h**eavy **m**etal **t**olerance factor 1)], are acutely required for the detoxification of heavy metals in different species including *Schizosaccharomyces pombe*, *Chlamydomonas reinhardtii*, *Caenorhabditis elegans*, *Drosophila melanogaster*, *Rattus norvegicus*, and *Homo sapiens* (Ortiz et al., 1992; Hanikenne et al., 2005; Vatamaniuk et al., 2005; Paterson et al., 2007; Sooksa-Nguan et al., 2009; Schwartz et al., 2010). For example, ABCB6/HMT-1 from *C. elegans* detoxifies Cd, copper (Cu), and As and is expressed in liver-like cells, the coelomocytes, as well as head neurons and intestinal cells, which are the cell types that are affected by heavy metal poisoning in humans (Vatamaniuk et al., 2005; Schwartz et al., 2010). It has been suggested that human ABCB6/HMT-1 transports the precursors of heme synthesis into the mitochondria (Krishnamurthy et al., 2006). However, subsequent studies have challenged this suggestion and thus the physiological substrate of ABCB6/HMT-1 protein is unknown.

The eukaryotic ABC transporters are organized either as “full-molecule” transporters consisting of two polytopic transmembrane domains (TMD1 and TMD2) and two ATP-binding domains [nucleotide-binding domain (NBD)1 and NBD2], or as half-transporters, containing one TMD and one NBD (Dean et al., 2001a,b; Paumi et al., 2009). In ABCB6/HMT-1-like proteins, one TMD is fused with one NBD to form a “half-molecule” ABC transporter (Vatamaniuk et al., 2005; Rea, 2007). Evidence from the X-ray structure analysis of bacterial ABC proteins shows that at least two NBDs are required to form a functional ABC transporter (Smith et al., 2002; Rees et al., 2009; Beis, 2015; Boswell-Casteel et al., 2017). This configuration is necessary for the binding and hydrolysis of ATP to mediate ATP-powered translocation of substrates across the lipid bilayer (Smith et al., 2002; Rees et al., 2009; Beis, 2015; Boswell-Casteel et al., 2017). Consistent with this rule, our past findings revealed that ABCB6/HMT-1 of *C. elegans* at a minimum homodimerizes (Kim et al., 2010).

**FIGURE 1:**
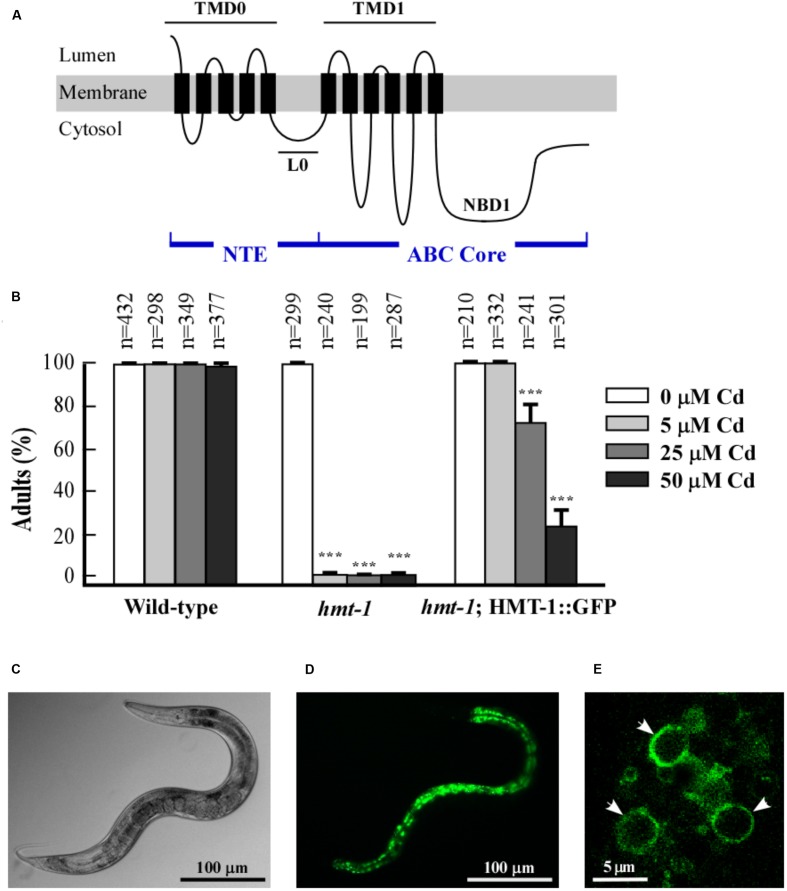
ABCB6/HMT-1 is expressed in intestine. **(A)** The predicted topology of half-ABC transporter ABCB6/HMT-1. The NTE- and ABC-core domains are indicated. The NTE domain of ABCB6/HMT-1 consists of five transmembrane spans (TMD_0_) and a cytosolic linker (L_0_), which connects the TMD_0_ with the ABC core domain consisting of TMD1 and NBD1. **(B)** The translational ABCB6/HMT-1::GFP construct rescues the Cd sensitivity of *hmt-1* mutant worms. The total number of worms tested (*n*) is written above each bar. Error bars indicate SDs from the mean values. Asterisks (^∗∗∗^*p* < 0.001) represent statistically significant differences between worms grown without vs. with CdCl_2_. **(C,D)** Representative DIC and fluorescence micrograph, respectively, of the pattern of ABCB6/HMT-1::GFP expression in the *hmt-1* mutant. **(E)** A confocal microphotograph of the subcellular localization of HMT-1::GFP expressed in the *hmt-1* mutant; arrow heads indicate that HMT-1::GFP localizes to the periphery of vesicular compartments.

In addition to the four-domain structure in full-transporters or two-domain structure in half-transporters, some ABC transporters possess a hydrophobic N-terminal extension domain termed NTE (Higgins, 1992; Rea, 2007; **Figure 1A**). The NTE domain consists of five to six transmembrane spans (TMD_0_) and a cytosolic linker (L_0_), which connects the TMD_0_ with core domains of ABC transporters (TMDs or NBDs).

The NTE domain is present in full-molecule ABC transporters, ABCC1/MRP1, ABCC2/MRP2 and Ycf1p, and in half-transporters, ABCB6/HMT-1 (Higgins, 1992; Vatamaniuk et al., 2005; Rea, 2007) (**Figure 1A**). Structure-function analyses established that the NTE domains of YCF1p and ABCC2/MRP2 play an important role in membrane trafficking, whereas the NTE domain and the COOH-terminal region of ABCC1/MRP1 contain redundant trafficking signals, which only become essential when one or the other region is missing or is mutated (Mason and Michaelis, 2002; Westlake et al., 2005). The NTE domains of two ABCB-family half transporters from *C. elegans*, HAF-4 and HAF-9, are dispensable for localization and function (Tanji et al., 2017). Studies using K562 cell line have shown that the NTE domain of human ABCB6/HMT-1 is important for localization but not for dimerization, membrane insertion and ATP binding/hydrolysis (Kiss et al., 2015). By contrast, we showed previously that the NTE domain of *C. elegans* ABCB6/HMT-1 is essential for its ability to form homodimers and ABCB6/HMT-1 functions in Cd resistance in the heterologous system (Kim et al., 2010). The role of the NTE domain in the function of ABCB6/HMT-1 in the native organism remains to be investigated.

A subcellular localization of ABCB6/HMT-1 proteins remains a matter of debate as well. For example, human ABCB6 (alias MTABC3) was initially identified in the mitochondria (Mitsuhashi et al., 2000). Subsequent studies have refined its localization to the outer mitochondrial membrane and this protein was found at the plasma membrane and lysosomes (Mitsuhashi et al., 2000; Paterson et al., 2007; Jalil et al., 2008; Paumi et al., 2009; Kiss et al., 2015; Boswell-Casteel et al., 2017). Kiss et al. (2012) have shown that the endogenous ABCB6 is localized to the endo/lysosomal compartment, and is absent from the mitochondria of cells. It is noteworthy, however, that the analyses of the subcellular localization of human ABCB6/HMT-1 were performed in heterologous systems, mostly in cell cultures (Boswell-Casteel et al., 2017). These studies did not resolve whether variation in the localization pattern reflect artifacts of the robust overexpression of a “tagged” ABCB6 and whether the “tagged” ABCB6 was, in fact, functional.

In this regard, *C. elegans* is a non-mammalian model system that provides the advantage of tinkering these questions in the intact multicellular organism. Thus, here, we used *C. elegans* to determine the subcellular localization of ABCB6/HMT-1. We also continued the structure-function studies of ABCB6/HMT-1 in *C. elegans* with the goal of identifying other functional regions in the polypeptide that play a role in localization, interactions, and ability to confer Cd resistance. We show that *C. elegans* ABCB6/HMT-1 localizes to the endosomal recycling system in intestinal cells, while the ABCB6/HMT-1 lacking the NTE domain tends to associate with the plasma membrane. We also show that ABCB6/HMT-1 lacking the NTE domain exerts a dominant negative effect on the localization and function of the full-length ABCB6/HMT-1 polypeptide suggesting that the C-terminal part of the protein is involved in protein–protein interactions as well. Thus, here we also identified the C-terminus region of HMT-1 that is necessary for ABCB6/HMT-1 dimerization and function in Cd resistance.

## Materials and Methods

### *Caenorhabditis elegans* Strains and Growth Culture Condition

*Caenorhabditis elegans* strains used in this study are listed in **Table 1**. Worms were maintained at 20°C on Solid Nematode Growth medium (NGM) using the Escherichia *coli* OP50 strain as a food source (Brenner, 1974). Males of VF12 *C. elegans* strain expressing translational HMT-1::GFP fusion were crossed using standard genetic procedures (Brenner, 1974) into strains expressing red fluorescent protein (RFP)- or mCherry-tagged markers for different endocytic compartments.

**Table 1:** List of worm strains used in this study.

Strains	Relevant genotype	Source/reference
N2	Wild-type	Schwartz et al., 2010
VF3	*hmt-1(gk161)III*	Schwartz et al., 2010
VF12	*hmt-1(gk161)III;gfIs1[phmt-1::hmt-1::gfp, unc-119(+)]*	Kim et al., 2010; Schwartz et al., 2010
VF13	*hmt-1(gk161);gfIs1[phmt-1-::hmt-1::gfp, unc119(+)]; pwIs428[vha-6p-rfp-rab-11, Cbunc-119(+)]*	In this study
VF23	*hmt-1(gk161)III; gfIs2[phmt-1::no nte-hmt-1::gfp]*	In this study
VF24	*hmt-1(gk161)III; gfEx4[phmt-1-nte::gfp]*	In this study
VF 25	*hmt-1(gk161)III; gfIs2[phmt-1::no nte-hmt-1::gfp]; pwIs428[pvha-6::rfp::-rab-11, Cbunc-119(+)]*	In this study
VF 26	*hmt-1(gk161)III; gfEx4[phmt-1-nte::gfp];pwIs428[pvha-6::rfp::-rab-11, Cbunc-119(+)]*	In this study
VF31	*gfIs1[phmt-1::hmt-1::gfp]*	In this study
VF32	*gfEx4 [phmt-1::nte::gfp]*	In this study
VF33	*gfIs2[phmt-1::no nte-hmt-1::gfp]*	In this study
VF37	*hmt-1(gk161)III; gfEx5[phmt-1::hmt-1::rfp]*	In this study
VF38	*hmt-1(gk161)III; gfIs1[phmt-1::hmt-1::gfp, unc-119(+)];gfEx5[phmt-1::hmt-1::rfp]*	In this study
VF39	*hmt-1(gk161)III; gfIs2[phmt-1::no nte-hmt-1::gfp];gfEx5[phmt-1::hmt-1::rfp]*	In this study
VF40	*hmt-1(gk161)III; gfEx4[phmt-1::nte::gfp]; gfEx5[phmt-1::hmt-1::rfp]*	In this study
VF46	*hmt-1(gk161)III; gfEx6[phmt-1::Δ758-801-hmt-1::gfp]*	In this study
VF48	*hmt-1(gk161)III; gfEx6[phmt-1::Δ758-801-hmt-1::gfp]; gfEx5[phmt-1::hmt-1::rfp]*	In this study
VF50	*gfEx6[phmt-1::Δ758-801-hmt-1::gfp]*	In this study
VF 57	*hmt-1(gk161)III;[plmn-1p::lmn-1::gfp::lmn-1 3’utr + pMH86; dpy-20(+)]*	In this study


### Plasmid Construction

A 10 kb genomic fragment, consisting of the promoter and the genomic sequence of *C. elegans hmt-1* was PCR-amplified and fused at the C-terminus with GFP of the *pPD117.01* vector (Fire et al., 1990) to generate the full-length *pPD117.01-ghmt-1::GFP* construct. To enable the cloning of ABCB6/HMT-1 truncated variants by recombination, the *pPD117.01-phmt-1::GFP* (Schwartz et al., 2010) was modified into the Gateway destination vector using the Gateway^®^ Vector Conversion System (Invitrogen). Briefly, the RfA (reading frame cassette A) was inserted into the *Age*I restriction site localized after the *phmt-1* and prior to 5′ of the GFP in the *pPD117.01-phmt-1::GFP*. The newly generated vector was designated *pPD117.01-phmt-1::GFP-Gate.* To enable co-localization studies, an open reading frame of RFP was PCR amplified using the primer pairs with engineered *Age*I and *Nhe*I restriction enzymes recognition sites at the 5′ and 3′, respectively (**Table 2**). The resulting PCR fragment was cloned into *Age*I and *Nhe*I sites of the *pPD117.01-phmt-1::GFP-Gate* to replace GFP with RFP and generate *pPD117.01-phmt-1::RFP-Gate.* Truncated *hmt-1* fragments (ΔNTE-HMT-1::GFP, NTE::GFP, or HMT-1^Δ43^::GFP, **Figure 4**) were generated by PCR using primer pairs listed in **Table 2** and introduced into the *pDONR222* entry vector before recombination with *pPD117.01-phmt-1::GFP-Gate* or *pPD117.01-phmt-1::RFP-Gate.*

**Table 2:** Primer list used in this study.

Variants	Primer sequence
HMT-1	FW: 5′-aca agt ttg tac aaa aaa gca ggc tct cca acc acc ATG GGC TTT TCA CCA TTT CTC GA-3′
	RV: 5′-tcc gcc acc acc aac cac ttt gta caa gaa agc tgg gta CGG AAG CTC CTC GCC GAG TTC AA -3′
ΔNTE-HMT-1	FW: 5′- aca agt ttg tac aaa aaa gca ggc tct cca acc acc ATG CAA CTT CGC GTC GTT TTT TG -3′
	RV: 5′- tcc gcc acc acc aac cac ttt gta caa gaa agc tgg gta CGG AAG CTC CTC GCC GAG TTC AA -3′
NTE	FW: 5′- aca agt ttg tac aaa aaa gca ggc tct cca acc acc ATG GGC TTT TCA CCA TTT CTC GA -3′
	RV: 5′- tcc gcc acc acc aac cac ttt gta caa gaa agc tgg gta GAGGGAAATTGATTTTGTCG -3′
HMT-1^Δ43^	FW: 5′- aca agt ttg tac aaa aaa gca ggc tct cca acc acc ATG GGC TTT TCA CCA TTT CTC GA -3′
	RV: 5′- tcc gcc acc acc aac cac ttt gta caa gaa agc tgg gta ATC AAG AAC AAG AAT AAG GTC -3′
RFP	FW: 5′- CCG ACCGGT ATGGCCTCCTCCGAGGACGT-3′
	RV: 5′- GTAGCTAGC TTAGGCGCCGGTGGAGTGGC-3′
*gk161* allele genotype	FW: 5′- AAATGGCGTAATCAACCGAG- 3′
	RV: 5′-TGAGCGGTGTGTAGAGTTGG-3′


### Generation of Transgenic Worms

To initiate structure-function analysis, we injected 100 ng/μl of *pPD117.01-Gate* carrying ΔNTE-HMT-1::GFP or NTE::GFP, or HMT-1^Δ43^::GFP into the gonadal syncytium of adult hermaphrodites of *hmt-1*(*gk161)* mutant worms to generate VF23, VF24, and VF46 strains, respectively (**Table 1**). We also injected *pPD117.01-phmt-1*; *hmt-1::rfp* into the *hmt-1*(*gk161)* mutant to create *VF37* worm strain expressing the full-length HMT-1::RFP fusion under the control of *hmt-1* promoter. N2 (wild-type) worms expressing the full-length HMT-1::GFP or ΔNTE-HMT-1::GFP, NTE::GFP, or HMT-1^Δ43^::GFP were generated by crossing with males of VF12, VF23, VF24, VF46 the newly generated strains were designated VF31, VF32 VF33, and VF50 (**Table 1**). Using genetic crosses, we also generated *hmt-1(gk161)* strains co-expressing ABCB6/HMT-1^Δ43^::GFP with the full-length ABCB6/HMT-1::RFP, both controlled by *hmt-1* promoter region (strain VF48, **Table 1**). VF57 was generated by crossing wild-type worms expressing LMN-1::GFP with VF3 (*hmt-1(gk161)*). We used Cd sensitivity assays to select for homozygous *hmt-1(gk161)* expressing different truncated or full-length GFP- or RFP-fusions. The genotype was confirmed by worm PCR. Briefly, 20 worms from each strain were collected into PCR lysis buffer containing 60 μg/ml proteinase K, 10 mM Tris-Cl, pH 8.2, 50 mM KCl, 2.5 mM MgCl_2_, 0.45% Tween-20, 0.05% gelatin and frozen at -80°C for 10 min to facilitate the release of genomic DNA (gDNA). A drop of mineral oil was placed on the top of the buffer prior to lysing worms for 1 h at 65°C followed by enzyme inactivation at 95°C for 15 min. The subsequent mixture containing gDNA served as a temple for PCR using primer pairs listed in **Table 2**. The expression of GFP or RFP was verified using a Leica MZ16FA automated fluorescence stereozoom microscope with the Leica EL6000 metal halide illuminator. To maintain stable inheritable expression of transgenes, the injected plasmids were integrated into the genome by γ-irradiation as described (Schwartz et al., 2010).

### Cadmium Sensitivity Assays

Cadmium sensitivity assays were performed as described (Schwartz et al., 2010). Briefly, two adult hermaphrodites were placed on solid NGM medium supplemented with indicated concentrations of CdCl_2_ and seeded with *E. coli* OP50. Worms were allowed to lay eggs for 4–5 h and adult worms were removed to obtain age-synchronized animals. The hatched worms were grown for 3–4 days (until the progeny of worms under control conditions have reached the adult stage). Cadmium sensitivity was assessed by comparing the percentage of progeny that have reached the adult stage under cadmium vs. control conditions. Morphological changes in intestinal cells were observed under the Zeiss Axio Imager M2 microscope. Results are presented as mean values from three independent experiments each of which had three replicates. The total number of worms tested (*n*) is presented above each graph bar.

### Imaging of the Subcellular Localization of ABCB6/HMT-1

**FIGURE 2:**
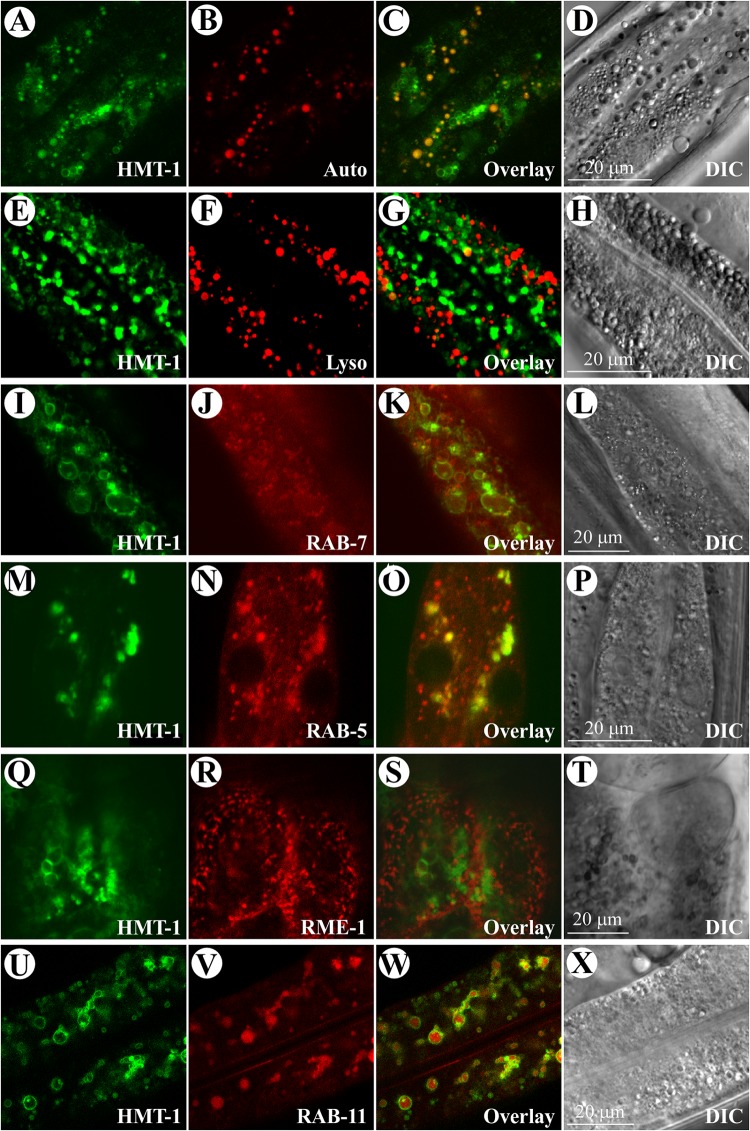
ABCB6/HMT-1::GFP resides on apically localized recycling endosomes of intestinal cells in *C. elegans*. **(A–D)** Confocal microscopy analysis of ABCB6/HMT-1::GFP and autofluorescence signals (Auto) in intestinal cells of the *hmt-1* mutant expressing ABCB6/HMT-1::GFP (HMT-1). Note that ABCB6/HMT-1::GFP-mediated fluorescence does not co-localize with the lysosome-related gut granules accumulating birefringent and autofluorescent material (Overlay). **(E–H)** *hmt-1* mutant worms expressing ABCB6/HMT-1::GFP were stained with the lysosomal dye, LysoTracker Red. Overlay image (Overlay) shows that the ABCB6/HMT-1::GFP fluorescence signal does not co-localize with the lysotracker-signal in lysosomes (Lyso). **(I–L)** Analysis of ABCB6/HMT-1 localization in the *hmt-1* mutant co-expressing ABCB6/HMT-1::GFP with a marker for early and late endosomes, RAB-7::RFP. The expression of RAB-7::RFP in intestinal cells was driven by the intestine-specific *vha-6* promoter. Overlay microphotograph (Overlay) shows that ABCB6/HMT-1::GFP fluorescence signal partially co-localizes with the RAB-7::RFP signal (RAB-7). **(M–P)** Analysis of ABCB6/HMT-1 localization in the *hmt-1* mutants co-expressing ABCB6/HMT-1::GFP with a marker for early endosomes, RAB-5::RFP. Overlay microphotograph (Overlay) shows that ABCB6/HMT-1::GFP fluorescence signal partially co-localizes with RAB-5::RFP signal (RAB-5). (**Q–T)** Analysis of HMT-1 localization in the *hmt-1* mutants co-expressing ABCB6/HMT-1::GFP with a marker of basolateral recycling endosomes, RME-1::RFP. Overlay microphotograph (Overlay) shows that ABCB6/HMT-1::GFP fluorescence signal does not co-localize with RME-1::RFP signal (RME-1). **(U–X)** Analysis of HMT-1 localization in the *hmt-1* mutants co-expressing ABCB6/HMT-1::GFP with a marker of apically located recycling endosomes, RAB-11::mCherry. Overlay microphotograph (Overlay) shows that ABCB6/HMT-1::GFP signal co-localizes with RAB-11::mCherry signal (RAB-11). A DIC microphotograph shows a section of the analyzed intestinal cells (DIC).

The initial characterization of the full-length ABCB6/HMT-1::GFP localization was done using the Zeiss LSM 710 confocal microscope system and ZEN 2009 software (Carl Zeiss MicroImaging, Cornell University Institute of Biotechnology). Live worms (young adults) were mounted on 2% agarose pads and immobilized with 20 mM sodium azide as described previously (Schwartz et al., 2010). To examine if ABCB6/HMT-1-GFP co-localizes with lysosome-related autofluorescent gut granules (Schroeder et al., 2007), GFP was excited with the 488 nm line of an Argon laser and detected via a 505–530 nm band pass filter. Autofluorescent gut granules were excited by the 543 nm laser line of a HeNe laser and detected with a 560 nm long pass filter. Fluorescent signals were acquired for both channels simultaneously. Having determined that ABCB6/HMT-1::GFP^-^mediated fluorescence does not co-localize with autofluorescent gut granules (**Figures 2A–D**), we established the emission peaks of GFP and gut granules autofluorescence. We determined that when excited with the 488 nm laser line, GFP emission peaked at 517 nm, whereas autofluorescence emission peaked at 530 nm. Therefore, to eliminate contributions from autofluorescent lysosome-related gut granules, in subsequent studies we collected GFP signal at 517 nm. In addition, to eliminate crosstalks between the emission from GFP and autofluorescence, or GFP and lysosomal dye, Lysotracker Red or GFP and RFP signals, we used 2-channel fast line-by-line multitracking technique.

To label lysosomes with LysoTracker Red (Molecular Probes, Eugene, OR, United States), L4 stage HMT-1::GFP expressing worms were placed on OP50 seeded NGM plates containing 4 μM LysoTracker Red. Animals were grown for 20 h without exposure to light before young adults were removed and examined by confocal microscopy. To determine whether HMT-1-GFP co-localizes with markers for different endocytic compartments, males of VF12 strain were crossed into worms expressing *rab-5::RFP* (early endosomal marker) or *rab-7::RFP* (late and early endosomal marker), or *rme-1::RFP* (basolateral recycling endosomes marker), or *rab-11:mCherry* (apical recycling endosomes marker) from the intestine-specific *vha-6* promoter (Oka et al., 2001; Su, 2005). These worm strains were the generous gift of Dr. Barth Grant, Rutgers University. Homozygous worms that did not segregate for GFP or DrRED were selected using Leica MZ16FA automated fluorescence stereozoom microscope with Leica EL6000 metal halide illuminator (Schwartz et al., 2010). The localization of GFP- and RFP-derived signals was examined by confocal microscopy using 2-channel fast line-by-line multitracking technique at 488 Ex/517 Em (for GFP) and 543 Ex/560Em (for RFP) laser lines.

After establishing the localization of the full-length ABCB6/HMT-1, the subsequent studies were done using Zeiss Axio Imager M2 microscope equipped with the motorized Z-drive as well as FITC and Texas Red filter sets for capturing GFP, RFP, and mCherry-mediated fluorescence. Images were collected with AxioCam MR Camera and the Zeiss AxioVision 4.8 software.

### Preparation of Microsomal and Soluble Proteins From *C. elegans*

Age-synchronized young adult worms were used for the protein isolation and fractionation. To obtain the sufficient amount of synchronized worms, 100 adult worms were grown on 10 OP50 *E. coli* seeded NGM plates (100 mm × 15 mm; 10 worms/plate) under standard condition for 3.5 days (until the progeny of inoculated worms have reached the adult stage and sufficient amount of embryos were visible on plates). The adult worms and eggs were washed with M9 buffer and collected by centrifugation at 3,500 × *g*. Worm and embryo pellets were resuspended in 5 ml of alkaline hypochlorite solution (0.25 M KOH, 1.2% NaOCl) and incubated with frequent agitation for 3–10 min at room temperature. This procedure dissolved gravid adults but remained embryos intact. The release of embryos was observed under a dissecting microscope. When ∼90% of worms were dissolved, unaffected embryos were collected by centrifugation at 3,500 × *g*, the supernatant was aspirated and embryos were washed free from alkaline bleach by five rounds of centrifugation (3,500 × *g*)/resuspension in M9 buffer. The final embryo pellet was resuspended in 2–3 ml of sterile M9 buffer, embryos were allowed to hatch, and were synchronized at the L1 stage in M9 during incubation at 20°C for 18 h with shaking. Approximately 10,000 L1s were then inoculated onto 150 mm × 15 mm NGM plates (∼5,000 worms/plate) seeded with OP50 *E. coli*. Worms were grown for 2.5 days at 20°C until L1 became young adults.

Young adult hermaphrodites were collected from plates with M9 medium and washed free from *E. coli* by two rounds of centrifugation (3,500 × *g* for 2 min) and resuspension in M9 medium. To replace M9 medium with lysis buffer, worm pellet from the second centrifugation was resuspended in a lysis buffer containing 50 mM TRIS-HCl, pH 7.6, 2 mM 2-mercaptoethanol 1 mM phenylmethylsulfonyl fluoride, and 1 μg/ml each of leupeptin, aprotinin, and pepstatin. After centrifugation at 3,500 × *g* for 2 min, the final worm pellet was resuspended in the same lysis buffer (1/1.5 of V worms/V buffer ratio) and transferred into eppendorf tubes. Worms were then broken by sonication at 4°C in the lysis buffer and worm debris was cleared by low-speed centrifugation at 3,500 × *g* for 10 min. The supernatant, containing microsomal and soluble proteins was collected and subjected to ultracentrifugation at 100,000 × *g* for 1 h using Beckman bench-top ultracentrifuge. The supernatant, containing soluble proteins was collected, frozen in liquid N_2_ and kept at -80°C for subsequent manipulations. The microsomal pellet (membrane-bound vesicles of total cellular membranes), was washed, re-pelleted at 100,000 × *g* and reconstituted in the same lysis buffer. Membranes were fractionated by sucrose step density gradient centrifugation using 16, 22, 28, 34, 40% sucrose solutions prepared in the same lysis buffer. About 500 μg of total microsomal membrane protein were subjected to ultracentrifugation for 2.5 h at 100,000 × *g* using Beckman SW41Ti rotor. Protein fractions were collected from each sucrose interface and washed free of sucrose by three rounds of centrifugation (100,000 × *g* for 1 h)/resuspension in the lysis buffer. The resulting membrane fractions were frozen in liquid N_2_ and stored at -80°C.

### Protein Electrophoresis and Immunoblotting

SDS-polyacrylamide gel electrophoresis (SDS-PAGE) and immunoblot analyses were done as described (Kim et al., 2010). Briefly, 5 μg of proteins were resolved on 7% SDS-PAGE gel and electrotransferred to 0.2 μm nitrocellulose membrane using a wet tank transfer. Electrotransfer was performed for 18 h at 4°C at a constant current of 60 mA in a Towbin buffer containing 0.05% SDS. The filters were blocked and probed with primary anti-GFP (Covance) or anti-RME-1, or anti-LMP-1 monoclonal antibodies, or the anti-RFP polyclonal antibody (Pierce) all used at 1:1,000 dilution and with the secondary HRP-linked anti-mouse or anti-rabbit IgG antibody (1:10,000 dilution, GE Healthcare). The immunoreactive protein bands were visualized with LumiGLO Peroxidase Chemiluminescent Substrate Kit (KPL).

### Co-immunoprecipitation

The suspension of membrane proteins in the IP buffer (1% PFO, 50 mM Tris-Cl, 150 mM NaCl, pH7.4) was pre-cleared with Protein G beads (Santa Cruz Biotechnology, Inc.) for 1.5 h at 4°C, Protein G beads were spun down and the supernatant was incubated for 4 h at 4°C with anti-GFP conjugated beads (Santa Cruz Biotechnology, Inc.) in the IP buffer. Supernatant was removed by low-speed centrifugation, beads were washed with IP buffer by three rounds of centrifugation/resuspension, and proteins were eluted in Laemmli buffer (62.5 mM Tris-HCl, pH 6.8, 25% glycerol, 2% SDS, 0.01% Bromophenol Blue) and used for the immunoblot analysis.

### Yeast-Two-Hybrid Assay

Yeast-two-hybrid assays were performed using the mating-based split-ubiquitin system (mbSUS) as described (Kim et al., 2010). The NubG and CubPLV prey and bait vectors, THY.AP4 and THY.AP5 yeast strains were obtained from the depository at *Arabidopsis* Biological Resource Center http://www.arabidopsis.org/abrc/index.jsp. Interaction between bait and prey constructs were detected by growing yeast cells on SC medium lacking adenine, histidine, leucine, tryptophan. β-galactosidase assay was conducted to confirm interactions.

### Statistical Analysis

At least three independent experiments each of which had three replicates were performed in all cadmium sensitivity assays. The number of animals tested is indicated above each graph bar in figures. We utilized two-way ANOVA with Bonferroni post-test to evaluate the statistical significance for our data. The asterisk above the graph bar indicates *p*-values of the data. Specifically, ^∗^ indicates *p* < 0.05, ^∗∗^ indicates *p* < 0.01, and ^∗∗∗^ indicates *p* < 0.001.

## Results

### ABCB6/HMT-1 Resides on the Apical Recycling Endosomes in Intestinal Cells of *C. elegans*

Our past studies using the transcriptional *phmt-1::gfp* reporter construct have shown that *ABCB6/HMT-1* is expressed in multiple tissues of *C. elegans* including head neurons, intestinal cells, and coelomocytes (Schwartz et al., 2010). To establish the subcellular localization of ABCB6/HMT-1, we generated the translational ABCB6/HMT-1::GFP construct (*phmt-1::hmt-1::gfp*). To test whether the GFP-tagged ABCB6/HMT-1 is functional, we used the *hmt-1* (*gk161)* mutant allele (from here on *hmt-1*) and integrated the translation ABCB6/HMT-1::GFP construct into the *hmt-1* mutant genome. The *hmt-1::gfp* construct fully rescued the sensitivity of *hmt-1* mutants to low concentration of Cd (5 μM CdCl_2_) (**Figure 1B**). Nearly 80% of *hmt-1* worms were rescued by *hmt-1::gfp* when worms were grown at higher Cd concentration (25 μM CdCl_2_), while only 20% were rescued at 50 μM CdCl_2_ (**Figure 1B**). We concluded that *phmt-1::hmt-1::gfp* is partially functional and proceeded with the analysis of its subcellular localization.

Confocal microscopy revealed that the bulk of ABCB6/HMT-1::GFP-mediated fluorescence was present in intestinal cells (**Figures 1C,D**) and was located at the periphery of the internal vesicular structures (**Figure 1E**). These vesicles were reminiscent of lysosomes and of the lysosome-related fat-storing gut granules that exhibit autofluorescence in GFP and rhodamine filter-sets (Hermann et al., 2005; Schroeder et al., 2007). To determine whether ABCB6/HMT-1::GFP also localized to gut granules, we compared fluorescence patterns of ABCB6/HMT-1::GFP and gut granules. We found that ABCB6/HMT-1::GFP-expressing vesicular structures were distinct from auto-fluorescent gut granules (**Figures 2A–D**). Therefore, to eliminate the contribution of autofluorescence, from then on we collected *hmt-1::GFP* signals at 517 nm as detailed in Section Materials and Methods. In addition, instead of using the classical way of acquiring the fluorescent signal for both, GFP and rhodamine channels simultaneously, we used 2-channel fast line-by-line multitracking technique. We then tested if ABCB6/HMT-1::GFP would localize to the lysosomes as does its homologs HsABCB6 and rAbcb6 from human and rats, respectively (Jalil et al., 2008; Kiss et al., 2015). We stained *hmt-1* mutants expressing ABCB6/HMT-1::GFP with a lypophilic, weakly basic red fluorescent dye, LysoTracker, that selectively accumulates in cellular compartments with low-internal pH such as vacuoles and lysosomes (Rodriguez-Enriquez et al., 2006; Ishida et al., 2013). We found that ABCB6/HMT-1::GFP was not associated with lysosomes (**Figures 2E–H**).

To establish the identity of ABCB6/HMT-1::GFP-localized subcellular organelles, we co-expressed this construct with marker proteins for different endocytic compartments (Campbell et al., 2002; Schroeder et al., 2007). To do so, we crossed males of VF12 strain into worms expressing either RAB-5p-RFP, or RAB-7p-RFP, or RME-1p-RFP, or RAB-11p-RFP and analyzed the resulting transgenic worms by confocal microscopy. We found that ABCB6/HMT-1::GFP did not co-localize with RME-1 (**Figures 2Q–T**) and only a minor fraction of ABCB6/HMT-1::GFP co-localized with markers for early and late endosomes, RAB-7 and RAB-5 (**Figures 2I–P**). SDS-PAGE and immunoblot analysis further confirmed that ABCB6/HMT-1 did not co-fractionate with lysosomal, basolateral recycling endosome or Golgi membrane markers (Supplementary Figure 1). By contrast, we observed a strict superimposition of the fluorescence from ABCB6/HMT-1::GFP and RAB-11::mCherry, the marker for the apical recycling endosomes (**Figures 2U–X**). Therefore, we concluded that ABCB6/HMT-1::GFP is expressed mainly in the apical recycling endosomes in *C. elegans* intestinal cells and, in part in early and late endosomes.

### ABCB6/HMT-1 Interacts With Itself in *C. elegans*

**FIGURE 3:**
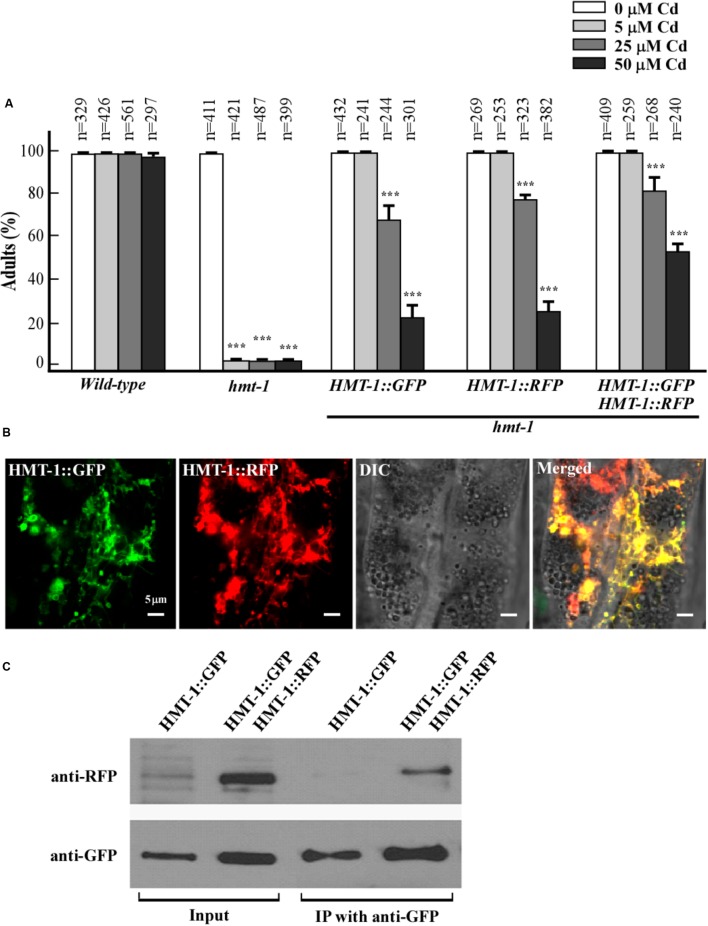
ABCB6/HMT-1 interacts with itself in *C. elegans*. **(A)** Cadmium sensitivity of wild-type, the *hmt-1* mutant, the *hmt-1* mutant expressing ABCB6/HMT-1::GFP (HMT-1::GFP) or ABCB6/HMT-1::RFP (HMT-1::RFP), and the *hmt-1* mutant co-expressing both, ABCB6/HMT-1::GFP and ABCB6/HMT-1::RFP constructs. The total number of worms tested (*n*) is indicated above each bar. Error bars show SD. Asterisks (^∗∗∗^*p* < 0.001) indicate statistically significant differences between worms grown without vs. with CdCl_2_. **(B)** A representative fluorescence microphotograph of the intestinal region of *hmt-1* mutants co-expressing ABCB6/HMT-1::GFP and ABCB6/HMT-1::RFP. **(C)** Co-immunoprecipitation assay shows that ABCB6/HMT-1::GFP interacts with ABCB6/HMT-1::RFP.

Our previous findings revealed that ABCB6/HMT-1 needs to interact with itself to confer Cd resistance in the heterologous system (Kim et al., 2010). Here, we asked whether ABCB6/HMT-1 interacts with itself in *C. elegans*. To test that we used co-immunoprecipitation and co-localization assays in *C. elegans*. We generated *hmt-1* mutants expressing ABCB6/HMT-1::RFP, and *hmt-1* mutants co-expressing ABCB6/HMT-1::GFP and ABCB6/HMT-1::RFP. We then evaluated whether ABCB6/HMT-1::RFP construct was functional by testing its ability to rescue the Cd sensitivity of the *hmt-1* mutant. We found that similar to ABCB6/HMT-1::GFP, the expression of ABCB6/HMT-1::RFP rescued the sensitivity of *hmt-1* mutant worms to low (5 μM) Cd and was also effective in rescuing the sensitivity to higher concentrations of Cd in the medium (**Figure 3A**). Co-expression of both ABCB6/HMT-1::GFP and ABCB6/HMT-1::RFP in the *hmt-1* mutant increased Cd tolerance of *hmt-1* worms even more (**Figure 3A**), and ABCB6/ HMT-1::RFP localized to the same endomembrane as ABCB6/HMT-1::GFP (**Figure 3B**). Therefore, animals expressing ABCB6/HMT-1::RFP or ABCB6/HMT-1::GFP or co-expressing both constructs were used to test whether ABCB6/HMT-1 interacts with itself in its native organisms. Based on the ability of the anti-GFP antibody to co-immunoprecipitate both ABCB6/HMT-1::GFP and ABCB6/HMT-1::RFP (**Figure 3C**), we concluded that ABCB6/HMT-1 interacts with itself in *C. elegans.* GFP has a tendency to dimerize (Tsien, 1998) suggesting that the observed protein–protein interactions of GFP-tagged ABCB6/HMT-1 could be an artifact of GFP dimerization. Our past studies of protein–protein interactions using yeast-two-hybrid system have shown that ABCB6/HMT-1 without the epitope tag interacts with itself and increases Cd tolerance of yeast cells (Kim et al., 2010). Therefore, we concluded that the observed protein–protein interaction of GFP-fused ABCB6/HMT-1 in the native organism is due to its ability to interact with itself and does not reflect a tendency of GFP to dimerize.

### The NTE Domain Is Essential for ABCB6/HMT-1 Function in Cadmium Resistance in *C. elegans*

**FIGURE 4:**
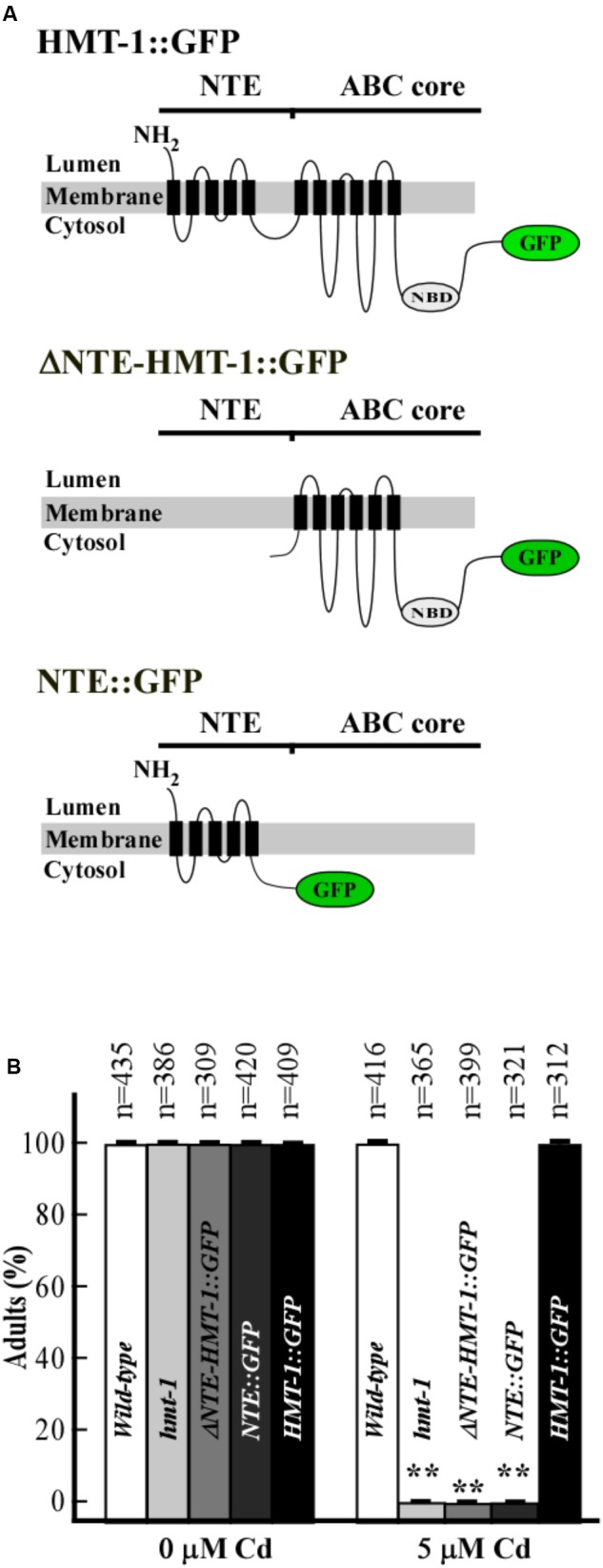
NTE is necessary but not sufficient for ABCB6/HMT-1-mediated Cd resistance. **(A)** Topology of ABCB6/HMT-1 variants: full-length ABCB6/HMT-1::GFP (HMT-1::GFP), ABCB6/HMT-1 lacking the NTE domain (ΔNTE-HMT-1*::*GFP), and the NTE domain of ABCB6/HMT-1 (NTE::GFP). **(B)** Cadmium sensitivity of wild-type, the *hmt-1* mutant and *hmt-1* mutant expressing indicated truncated variants of ABCB6/HMT-1. The total number of worms tested (*n*) is written above each bar. Error bars indicate SD. Asterisks (^∗∗^*p* < 0.01) show statistically significant differences between differences between worms grown without vs. with CdCl_2_.

The NTE domain was previously shown to play a critical role in Cd resistance of *S. cerevisiae* ABC transporter, Ycf1p (Mason and Michaelis, 2002). Also, our studies in yeast have shown the important role of the NTE in the function of ABCB6/HMT-1 in providing Cd resistance in the heterologous system (Kim et al., 2010). Here, we tested the role of the NTE domain in ABCB6/HMT-1 function and localization in *C. elegans*. To do so, in addition to the full-length ABCB6/HMT-1::GFP, we generated several ABCB6/HMT-1 variants: a construct lacking the NTE domain (ΔNTE-HMT-1::GFP) and a construct containing only the NTE domain (NTE::GFP) (**Figure 4A**). In all cases ABCB6/HMT-1::GFP variants were expressed under the control of the *hmt-1* promoter.

**FIGURE 5:**
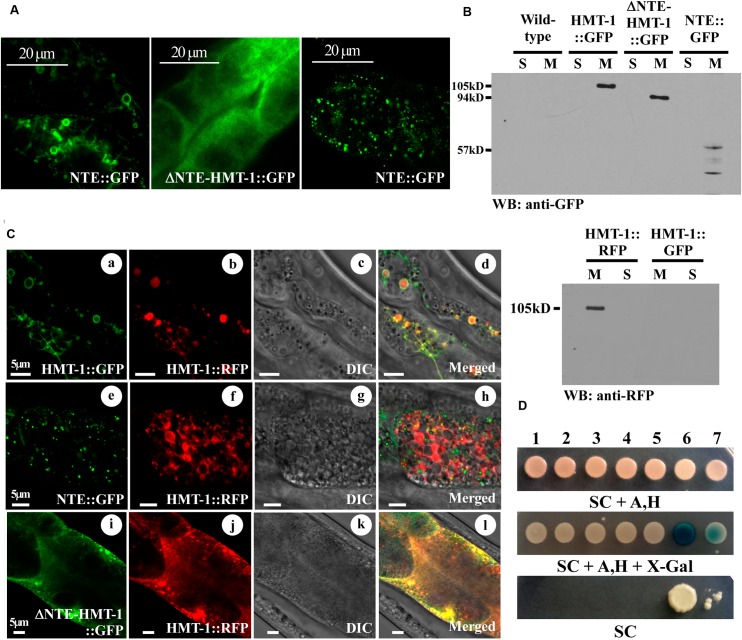
ΔNTE-HMT-1 affects the subcellular localization of the full-length ABCB6/HMT-1. **(A)** Representative image of GFP-mediated fluorescence in *hmt-1* mutants expressing the full-length ABCB6/HMT-1::GFP (Left) or ΔNTE-HMT-1::GFP (Middle) or NTE::GFP (Right). **(B)** Western blot analysis of soluble (S) and membrane (M) fractions of proteins isolated from wild-type worms or *hmt-1* mutant expressing full-length ABCB6/HMT-1::GFP (HMT-1::GFP), ΔNTE-HMT-1::GFP (ΔNTE-HMT-1::GFP), NTE::GFP (NTE) or full-length ABCB6/HMT-1::RFP (HMT-1::RFP). **(C)** Representative image of *hmt-1* mutants co-expressing full-length ABCB6/HMT-1::RFP with ABCB6/HMT-1::GFP **(a–d)** or with NTE::GFP **(e–h)** or with ΔNTE-HMT-1::GFP **(i–l)**. Full-length ABCB6/HMT-1::GFP co-localizes with ABCB6/HMT-1::RFP **(a–d)**. The majority of ABCB6/HMT-1::RFP co-localizes with ΔNTE-HMT-1::GFP at the plasma membrane **(i–l)**. NTE::GFP does not co-localize with full-length ABCB6/HMT-1::RFP **(e–h)**. **(D)** The mbSUS yeast-two-hybrid assay shows that ABCB6/HMT-1 lacking the NTE weakly interacts with the full-length ABCB6/HMT-1. Numbers indicate the following combination of constructs **1.** Empty vector::Cub + ΔNTE-HMT-1::NubG, **2.** KAT-1::Cub + ΔNTE-HMT-1::NubG, **3.** KAT-1::Cub + NTE::NubG, **4.** KAT-1::Cub + HMT-1::NubG, **5.** HMT-1::Cub + NTE::NubG, **6.** HMT-1::Cub + HMT-1::NubG, **7.** HMT-1::Cub + ΔNTE-HMT-1::NubG. KAT-1, potassium channel from *A. thaliana*, is used as a negative control. Shown are representative results of at least three biological replicates. Interactions were manifested by the ability of cells to grow on SC media without adenine and histidine (SC), and appearance of blue color, the product of β-galactosidase activity in the medium with X-gal (SC, Δsynthetic complete medium; Ade, Δadenine; His, Δhistidine; X-gal, 5-bromo-4-chloro-3-indolyl-β-D-galactopyranoside). Serial dilutions of yeast expressing bait and prey constructs were as indicated.

We first determined the ability of truncated ABCB6/HMT-1 proteins to rescue the Cd sensitivity of the *hmt-1* mutants. We found that while wild-type and *hmt-1* mutant worms expressing the full-length ABCB6/HMT-1::GFP were not sensitive to 5 μM CdCl_2_ (**Figure 4B**), the *hmt-1* mutant expressingABCB6/HMT-1::GFP without the NTE domain, or only the NTE domain were as sensitive to 5 μM CdCl_2_ as *hmt-1* mutant worms (**Figure 4B**). The truncated proteins were expressed and were associated with the membrane fraction of proteins in *C. elegans* as evident by the immunoblot analysis (**Figure 5B**). These results are consistent with our past findings in *S. cerevisiae* (Kim et al., 2010) and substantiate the conclusion that the NTE domain is essential but not sufficient for the function of ABCB6/HMT-1 in Cd resistance.

### The NTE Domain Is Essential but Not Sufficient for the Subcellular Localization of ABCB6/HMT-1

The NTE domain of ABCB6 in humans plays a role in targeting the protein to the lysosomal membrane (Kiss et al., 2015). Similarly, the NTE domain was previously shown to play a critical role in membrane trafficking of ABC transporter family members such as Ycf1p in yeast and MRP1/ABCC1 in mammalian cells (Mason and Michaelis, 2002; Westlake et al., 2005). We, therefore, suggested that ABCB6/HMT-1 lacking the NTE domain would be mislocalized and thus would not be able to rescue the Cd sensitivity of *hmt-1* mutant worms. To test this prediction, we compared the localization pattern of the full-length functional ABCB6/HMT-1::GFP, ΔNTE-HMT-1::GFP, and NTE::GFP, which were individually expressed in *hmt-1* mutant worms. Although the localization of ΔNTE-HMT-1::GFP expression was rather diffuse, the significant component of it was concentrated toward the plasma membrane of intestinal cells (**Figures 5A,C**). To ensure that the diffuse distribution of fluorescence was not due to the degradation of ΔNTE-HMT-1::GFP that would lead to cytosolic localization of GFP, total cellular lysates from the *hmt-1* mutants expressing ABCB6/HMT-1::GFP, or ΔNTE-HMT-1::GFP, or NTE::GFP were fractioned into soluble and membrane protein fractions and subjected to SDS-PAGE and immunoblot analysis. These studies showed that all fusion protein variants were associated with the membrane fraction of proteins and thus, the ΔNTE-HMT-1::GFP or NTE::GFP constructs did not degrade (**Figure 5B**).

In contrast to ΔNTE-HMT-1::GFP, the NTE::GFP-mediated fluorescence was associated with internally located vesicular structures and, in part, with the plasma membrane. However, these structures did not resemble the localization pattern of the full-length ABCB6/HMT-1::GFP (**Figures 5A,C**). To test whether HMT-1 variants are associated with the apical recycling endosome, we co-expressed truncated ABCB6/HMT-1::GFP variants with a marker for apical recycling endosomes, RAB-11::mCherry. We found that ΔNTE-HMT-1::GFP and NTE::GFP did not co-localized with RAB-11::mCherry (Supplementary Figure 2). Together, these results suggested that the NTE domain is essential but not sufficient for guiding ABCB6/HMT-1 to recycling endosomes.

### ΔNTE-HMT-1 Alters the Localization and Function of the Full-Length ABCB6/HMT-1

Since the NTE domain is essential but is not sufficient for the interaction and cellular localization of ABCB6/HMT-1, we next sought to determine whether its C-terminal part has a role in HMT-1–HMT-1 protein interaction, cellular localization, and function. To do so, we co-expressed ΔNTE-HMT-1::GFP or NTE::GFP HMT-1 with the full-length ABCB6/HMT-1::RFP in the *hmt-1* mutant and examined the subcellular localization and the functional activity of the full-length ABCB6/HMT-1::RFP. We found that the co-expression of the full-length ABCB6/HMT-1::RFP with NTE::GFP did not significantly change the localization pattern of the full-length polypeptide. The bulk of HMT-1::RFP-mediated fluorescence was associated with the apical recycling endosomes and only a minor fraction overlapped with NTE::GFP and was located to the internal vesicular structures (**Figure 5C**, e–h). Unexpectedly, the co-expression of the ΔNTE-HMT-1::GFP with the full-length HMT-1::RFP altered the pattern of the expression of the full-length polypeptide (**Figure 5C**). Specifically, while the full-length ABCB6/HMT-1 localized to the internal vesicular structures that corresponded to apical recycling endosomes, regardless whether it was tagged with the RFP or GFP (**Figures 2**, **3**, **5C**, a–d), the full-length ABCB6/HMT-1::RFP was predominantly localized to the plasma membrane when it was co-expressed with the ΔNTE-HMT-1::GFP (**Figure 5C**, i–l). This result suggested that two constructs may have interacted *in vivo* and that this interaction altered their localization pattern compared to the localization pattern of individually expressed constructs (**Figures 5A,C**). This suggestion is consistent with our finding that ΔNTE-HMT-1 weakly interacts with the full-length ABCB6/HMT-1 in yeast-two-hybrid assays (**Figure 5D**) (Kim et al., 2010).

**FIGURE 6:**
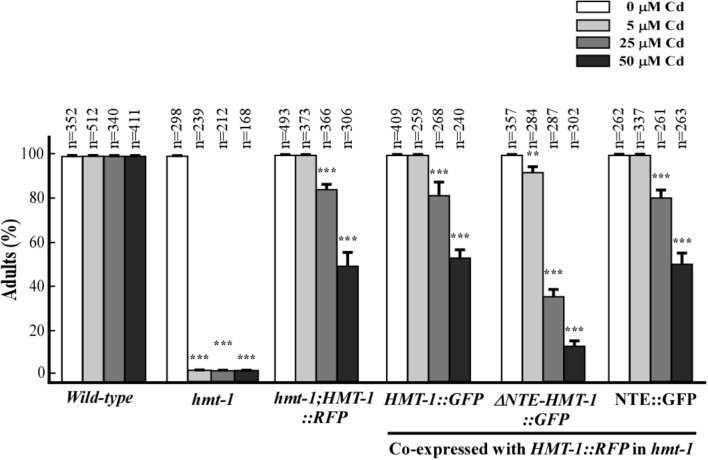
ΔNTE-HMT-1 exerts a dominant negative effect on the function of the full-length ABCB6/HMT-1 in Cd resistance. Wild-type worms or the *hmt-1* mutant expressing the indicated constructs when grown in the presence of the indicated concentrations of cadmium (Cd). Cd sensitivity is expressed as the percentage of worms that have reached the adult stage of the development. The total number (*n*) of worms tested is shown above each bar. The error bar shows SD. Asterisks (^∗∗^*p* < 0.01, ^∗∗∗^*p* < 0.001) indicate statistically significant differences between worms grown without vs. with CdCl_2_.

Given that correct cellular localization of proteins are essential for their function, we predicted that ΔNTE-HMT-1::GFP will also affect the ability of full-length ABCB6/HMT-1 to confer Cd resistance. To test this hypothesis, we analyzed Cd sensitivity of transgenic *hmt-1* mutant worms co-expressing the full-length ABCB6/HMT-1::RFP with ABCB6/HMT-1::GFP variants. We found that the co-expression of the full-length ABCB6/HMT1::RFP with the NTE::GFP rescued the Cd-sensitivity of the *hmt-1* mutant (**Figure 6**). This finding is consistent with the minor effect of the NTE::GFP on the expression pattern of ABCB6/HMT-1::RFP (**Figure 5**, e–h). By contrast, the co-expression of ABCB6/HMT-1::RFP with ΔNTE-HMT-1::GFP significantly altered the ability of the full-length polypeptide to rescue the Cd sensitivity of *hmt-1* mutant worms (**Figure 6**). Specifically, nearly all of *hmt-1* mutant worms co-expressing full-length ABCB6/HMT-1::RFP and ABCB6/HMT-1::GFP or *hmt-1* mutant worms co-expressing full-length ABCB6/HMT-1::RFP with NTE::GFP reached the adult stage in the presence of 25 μM of Cd. By contrast, only 38 ± 3.5%, of the *hmt-1* mutants co-expressing the ΔNTE-HMT-1::GFP with the full-length ABCB6/HMT-1::RFP reached the adult stage under this condition and only 8.3 ± 1.5% of worms have reached the adult stage under 50 μM of Cd (**Figure 6**). These findings suggested that ΔNTE-HMT-1 exerts a dominant negative effect on the full-length polypeptide. Consistent with this suggestion, the expression of ΔNTE-HMT-1::GFP in wild-type N2 worms significantly increased their Cd sensitivity (Supplementary Figure 3).

Our past studies have shown that the intestinal cells of Cd-grown *hmt-1* mutant worms exhibit characteristic morphological phenotypes manifested by the formation of refractile inclusions (Vatamaniuk et al., 2005). This phenotype is specific to *hmt-1* mutants and is not observed in any other Cd-sensitive mutants (Vatamaniuk et al., 2005 and data not shown). We, therefore, predicted that if ΔNTE-HMT-1 exerts a dominant negative effect on the function of ABCB6/HMT-1, but not on other ABC-transporters and their pathways *C. elegans*, then wild-types worms expressing ΔNTE-HMT-1::GFP would also develop refractile inclusion in intestinal cells in the presence of Cd.

**FIGURE 7:**
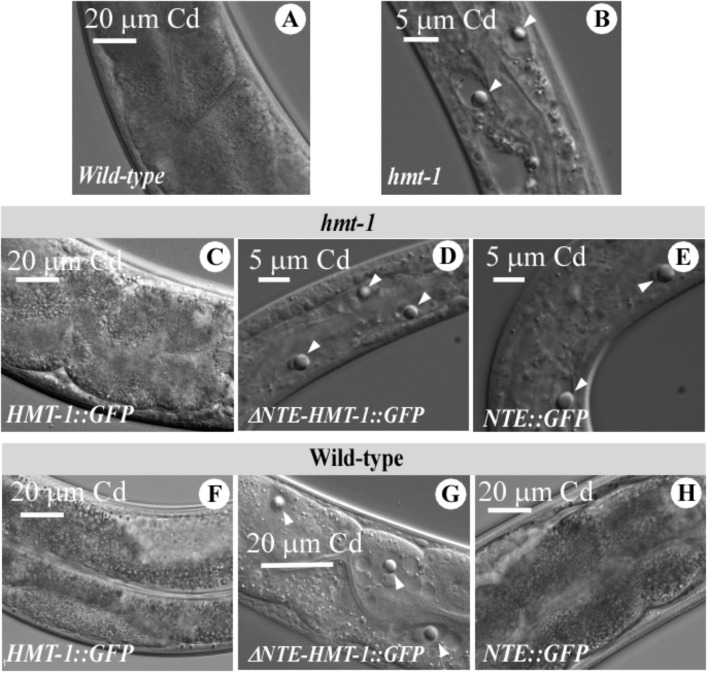
Refractile inclusions appear in Cd-grown wild-type worms expressing ΔNTE-HMT-1::GFP. A representative DIC image of wild-type **(A)**, *hmt-1* mutant worms **(B)** and the *hmt-1* mutant expressing either ABCB6/HMT-1::GFP **(C)**, or **Δ**NTE-HMT-1::GFP **(D)**, or NTE::GFP **(E)** and wild-type expressing either full length ABCB6/HMT-1::GFP **(F)**, or **Δ**NTE-HMT-1::GFP **(G)**, or NTE::GFP **(H)**. Adult hermaphrodites were placed individually onto NGM plates with the indicated concentrations of Cd and allowed to lay eggs. Images were collected after 4 days of growth at 20°C. The refractile inclusions are indicated with white arrows.

As we showed previously, refractile inclusions appeared in intestinal cells of Cd-grown *hmt-1* mutant worms but not in wild-type (**Figures 7A,B**) (Vatamaniuk et al., 2005). We also found refractile inclusions in intestinal cells of Cd-grown *hmt-1* mutant worms expressing the truncated ABCB6/HMT-1 constructs but not the full-length ABCB6/HMT-1 (**Figures 7C–E**). Refractile inclusions also appeared in wild-type worms expressing ΔNTE-HMT-1::GFP but not in worms expressing the full-length ABCB6/HMT-1::GFP or NTE::GFP only (**Figures 7F–H**). These results are consistent with the suggestion that the function of endogenous ABCB6/HMT-1, but not other ABC-transporter pathways or other metal-detoxification pathways, was disabled in wild-type worms co-expressing ΔNTE-HMT-1.

The nature of these retractile inclusions is unknown. We noticed, however, that these inclusions usually reside near the nucleus. To test whether indeed these inclusions are associated with the nucleus, we expressed the nuclear envelop marker, LMN-1::GFP marker that labels the nuclear envelope, in *hmt-1* mutant worms and exposed the transgenic worms to Cd. These analyses revealed that refractile inclusion indeed might be associated with the nucleus since they resided within the LMN-1::GFP marker (Supplementary Figure 4). Taken together, our findings suggest that ΔNTE-HMT-1 play a dominant negative effect on Cd detoxification function of ABCB6/HMT-1 through obstructing the localization of the full-length polypeptide to recycling endosomes. Most likely, the dominant negative effect is a result of the protein–protein interaction of ΔNTE-HMT-1 with the full-length polypeptide (**Figure 5D**). Thus, the C-terminal part of ABCB6/HMT-1 is also involved in ABCB6/HMT-1 protein interactions. Finally, our findings also show that in addition to lysosomes, the recycling endosomes are essential for Cd detoxification processes.

### The C-Terminus Is Important for ABCB6/HMT-1 Function an Ability to Interact With Itself

**FIGURE 8:**
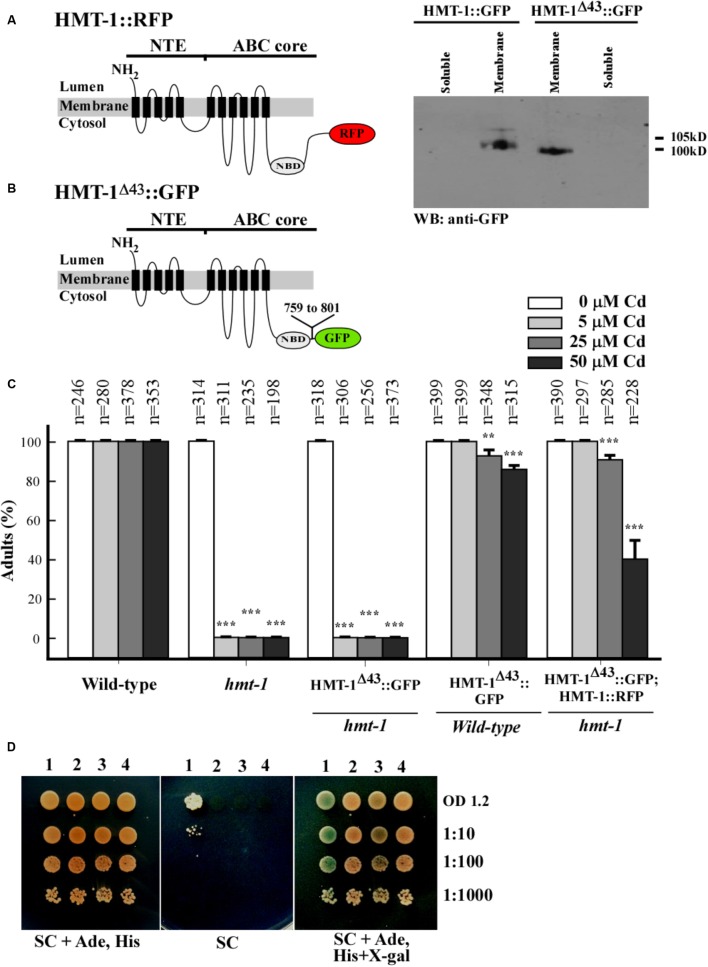
The C-terminus of ABCB6/HMT-1 contains protein motif(s) for ABCB6/HMT-1 interaction. **(A)** The topology of ABCB6/HMT-1::RFP and ABCB6/HMT-1^Δ43^::GFP. Position of the deletion of 43 amino residues (759-KGIILERGNHKELLAQQGTYASMWEAQIAEQRAKSIELGEELP-801) is indicated. Western blot analysis of HMT-1^Δ43^::GFP and full-length ABCB6/HMT-1::GFP shows that HMT-1^Δ43^::GFP co-fractions with the membrane fraction. **(C)** The percentages of wild-type worms or *hmt-1* mutant expressing the indicated constructs that had reached the adult stage when grown in the presence of the indicated concentrations of cadmium (Cd). The total number of worms tested (*n*) is shown above each bar. The error bar shows SD. Asterisks (^∗∗^*p* < 0.01, ^∗∗∗^*p* < 0.001) indicate statistically significant differences between the worms grown without vs. with CdCl_2_. Note that HMT-1^Δ43^::GFP failed to rescue Cd sensitivity of *hmt-1* mutant worms. The total number of worms tested (*n*) is provided above each bar. **(D)** Protein–protein interactions of ABCB6/HMT-1 as detected by the mbSUS yeast-two-hybrid assay. The number above the figures indicate the following combination of constructs: 1. ABCB6/HMT-1::Cub + HMT-1::NubG, 2. KAT-1::Cub + ABCB6/HMT-1::NubG, 3. ABCB6/HMT-1::Cub + ABCB6/HMT-1^Δ43^::NubG, 4. Empty vector::NubG + ABCB6/HMT-1::Cub. Yeast-two-hybrid assay shows that HMT-1^Δ43^ does not interact with the full-length HMT-1. KAT-1, potassium channel from *A. thaliana* was used as a negative control. Shown are representative results of at least three biological replicates. Interactions were manifested by the ability of cells to grow on SC media without adenine and histidine (SC), and appearance of blue color, the product of β-galactosidase activity in the medium with X-gal (SC, Δsynthetic complete medium; Ade, Δadenine; His, Δhistidine; X-gal, 5-bromo-4-chloro-3-indolyl-β-D-galactopyranoside). Serial dilutions of yeast expressing bait and prey constructs were as indicated.

To identify additional protein motif(s) in the ABC core region that might be involved in protein–protein interaction, we tested the function of the C-terminal end, in particular, 43 amino acid residues beyond the Walker A ATPase motif (759-KGIILERGNHKELLAQQGTYASMWEAQIAEQRAKSIELGEELP-801), because this part was shown to be essential for localization of ABCC1/MRP1 from humans (Westlake et al., 2005). We deleted 43 amino acid residues from the C-terminus of ABCB6/HMT-1 and then generated transgenic *hmt-1* mutant worms expressing ABCB6/HMT-1^Δ43^::GFP or *hmt-1* worms co-expressing ABCB6/HMT-1^Δ43^::GFP and full-length ABCB6/HMT-1::RFP in **Figure 8A**. Western blot analysis confirmed that ABCB6/HMT-1^Δ43^::GFP was expressed in worms (**Figure 8B**). We then tested whether ABCB6/HMT-1^Δ43^::GFP would rescue the Cd sensitivity of *hmt-1* mutant worms and found that *hmt-1* mutants expressing HMT-1^Δ43^::GFP were acutely sensitive to Cd (**Figure 8C**). These findings suggested that the 43 amino-acid region in the C-terminus of ABCB6/HMT-1 is required for its function in Cd resistance. However, unlike ΔNTE-HMT-1::GFP, ABCB6/HMT-1^Δ43^::GFP did not increase Cd sensitivity of wild-type or *hmt-1* mutant worms expressing full-length ABCB6/HMT-1::RFP (**Figure 8C**). This finding suggested that the modified polypeptide did not exert a dominant negative effect on the function of the full-length ABCB6/HMT-1 and that the deleted 43 amino acid residues might be needed for the protein–protein interactions. To test whether the inability of ABCB6/HMT-1^Δ43^::GFP to confer Cd resistance of the *hmt-1* mutants was due to a loss of ABCB6/HMT-1 homo-oligomerization, we conducted the yeast two-hybrid assay. The results revealed that ABCB6/HMT-1^Δ43^ does not interact with the full-length ABCB6/HMT-1 (**Figure 8D**). Together, our results indicate that the 43 amino-acid region in the C-terminus of ABCB6/HMT-1 is important for the function of ABCB6/HMT-1 in Cd resistance and the ability to interact with itself.

## Discussion

The ABC transporters are a conserved group of integral membrane proteins that mediate the Mg⋅ATP-energized transport of a wide range of substrates including anticancer drugs, cellular toxins, and heavy metals (Dean et al., 2001a,b; Rea, 2007; Wilkens, 2015). The functional “full-molecule” ABC transporters typically contain two TMD and two NBDs. The four-domain structure (two TMDs and two NBDs) that comprise a functional ABC transporter can be formed from a single polypeptide or by the association of two or four separate subunits (Higgins, 1992). In eukaryotes, most ABC proteins are encoded as single polypeptides containing two TMDs and two NBDs (Dean et al., 2001a,b). By contrast, half-molecule ABC transporters contain one TMD and one NBD, and function by forming homo- or hetero-oligomers. Some members of both categories possess a hydrophobic NTE, encompassing five to six TMDs and a cytosolic linker sequence contiguous with TMD or NBD (Higgins, 1992).

ABCB6/HMT-1 proteins from all species are half-molecule ABC transporters and can be distinguished from other half-transporters by the presence of the NTE domain, followed by a TMD fused to an NBD (Emadi-Konjin et al., 2002; Vatamaniuk et al., 2005; Sooksa-Nguan et al., 2009; Boswell-Casteel et al., 2017; **Figure 1A**). Studies of the functional significance of NTE showed that in some topologically similar full-molecule ABC transporters, NTE is required for proper membrane trafficking and oligomerization, whereas in others, acts redundantly with the COOH-terminal region and thus, is dispensable for membrane trafficking (Mason and Michaelis, 2002; Westlake et al., 2005). The role of the NTE domain in the function of ABCB6/HMT-1 in the native organism remains to be investigated.

ABCB6/HMT-1 proteins are acutely required for the detoxification of heavy metals in different species including humans (Ortiz et al., 1992; Hanikenne et al., 2001; Vatamaniuk et al., 2005; Paterson et al., 2007; Sooksa-Nguan et al., 2009). Their role in the detoxification processes suggested that they might be associated with lysosomes or their functional analogs, vacuoles (Blaby-Haas and Merchant, 2014; Polishchuk and Polishchuk, 2016). However, reports in the literature localize human ABCB6/HMT-1 to multiple membrane systems including outer mitochondrial membrane, plasma membrane, Golgi, ER, and the endosomal system (reviewed in Boswell-Casteel et al., 2017). ABCB6/HMT-1 of *S. pombe* localizes to the vacuolar membrane (Ortiz et al., 1995; Jalil et al., 2008). Our previous studies showed that heterologously expressed ABCB6/HMT-1 of *D. melanogaster* and *C. elegans* reside on the vacuolar membrane in *S. pombe* suggesting that in the intact organism they would be targeted to the vacuolar analogs, the lysosomes, as well (Vatamaniuk et al., 2005; Sooksa-Nguan et al., 2009). We note that all of these studies were done in heterologous systems and that the subcellular localization of ABCB6/HMT-1 proteins in its native organism has not been yet tested.

Here, we used a versatile multicellular organism, the nematode worm, *C. elegans*, to examine the subcellular localization of ABCB6/HMT-1 in its native organism. Specifically, we expressed ABCB6/HMT-1::GFP or RFP fusions under the control of the *hmt-1* promoter in the *hmt-1* mutant. Both constructs were functional since they rescued the Cd hypersensitive phenotype of *hmt-1* mutant worms (**Figures 1B**, **3A**) suggesting that translational ABCB6/HMT-1::GFP or RFP fused proteins reside at the correct endomembrane. We then found that the bulk of ABCB6/HMT-1::GFP or ABCB6/HMT-1::RFP fluorescence is associated with intestinal cells (**Figures 1C,D**, **3B**) and is localized to the periphery of intracellular vesicles that resembled lysosomes (**Figure 1E**). To our surprise, ABCB6/HMT-1::GFP did not associate with the lysosomes as evident by a distinct fluorescent pattern from ABCB6/HMT-1::GFP and a lysosomal dye, lysotracker (**Figures 2E–H**). The majority of ABCB6/HMT-1::GFP co-localized with the marker for apical recycling endosomes, RAB-11 (**Figures 2U–X**). We also found that a minor fraction of ABCB6/HMT-1::GFP was co-localized with markers for early and late endosomes, RAB7 and RAB5 (**Figures 2I–L,M–P**, respectively). Based on these results, we concluded that ABCB6/HMT-1 localizes to the apical recycling endosomes and, in part, to early and late endosomes in *C. elegans*. It is noteworthy that early, late, and recycling endosomes comprise the endosomal-recycling system that is involved in sorting, re-exporting, and degradation of membrane constituents and extracellular solutes including minerals and associated ligands (e.g., the Fe-transferrin complex) that are internalized via the endocytic uptake at the plasma membrane (Taguchi, 2013; Goldenring, 2015). Early recycling endosomes serve as hubs and modifiers of extracellular solutes (e.g., Fe stripping from the transferrin occurs in the early recycling endosomes). Early endosomes then direct these cargoes either to late endosomes and then after to lysosomes for degradation/recycling, or re-route these cargoes to recycling endosomes. Recycling endosomes, in turn, deliver these cargoes to the plasma membrane for exocytosis, or shunt them into autophagy pathways, or recycle them in lysosomes (Goldenring, 2015). It is unlikely that the localization of ABCB6/HMT-1 to recycling endosomes is a component of its trafficking to the plasma membrane because the plasma membrane-localized ABCB6/HMT-1 is unfunctional [**Figures 5C** (i–l), 6, and discussed below]. The fact that the functional ABCB6/HMT-1 localizes to recycling endosomes and, in part, to early and late endosomes suggests that the endosomal-recycling system in *C. elegans* is involved in Cd detoxification processes.

It is noteworthy that although ABCB6/HMT-1 proteins have a clear role in allowing organisms to survive Cd, their physiological substrates and function are not established (Vatamaniuk et al., 2005; Sooksa-Nguan et al., 2009). It has been suggested that human ABCB6/HMT-1 transports porphyrins into the mitochondria for the synthesis of the essential, Fe-binding molecule, heme (Krishnamurthy et al., 2006). However, subsequent studies have challenged the subcellular localization of ABCB6/HMT-1 and its contribution to the direct mitochondrial uptake of the intermediates of heme synthesis (Paterson et al., 2007; Jalil et al., 2008; Kiss et al., 2015). Furthermore, *C. elegans* is a heme auxotroph and relies on the dietary heme uptake and import into intestinal cells but not on the *de novo* synthesis in mitochondria (Rao et al., 2005; Sinclair and Hamza, 2015). Given that Cd interferes with the cellular uptake and binding of Fe to metalloenzymes and metal ligands (Eide et al., 1996; Park et al., 2002), it is tempting to speculate that *C. elegans* ABCB6/HMT-1 detoxifies Cd by eliminating toxic by-products of Cd-heme interactions into the endosomal-recycling system and, perhaps contributes to heme trafficking in the endocytic pathway. In this regard, a member of the full-molecule ABC transporter family, ABCC5/MRP-5 in *C. elegans* is a heme exporter that localizes to the secretory pathway and is involved in heme homeostasis (Korolnek et al., 2014).

We have also refined our past structure-function studies of ABCB6/HMT-1 in its native organism. Specifically, we showed that the NTE domain is essential but not sufficient for the localization and function of ABCB6/HMT-1. This conclusion was based on findings that the NTE domain alone or truncated ABCB6/HMT-1 lacking the NTE were not able to rescue the Cd sensitivity of the *hmt-1* mutant, and although truncated polypeptides were associated with the membrane fraction of proteins, they did not reside to the apical recycling endosomes in *C. elegans* (**Figures 4B**, **5A,B**). Furthermore, we found that the truncated ABCB6/HMT-1 polypeptide lacking the NTE domain exerted a dominant negative effect on the full-length ABCB6/HMT-1. This conclusion was based on finding that the full-length ABCB6/HMT-1 failed to rescue the Cd sensitivity of *hmt-1* mutant worms and mislocalized to the plasma membrane when it was co-expressed with ABCB6/HMT-1 lacking the NTE (**Figures 5C**, **6**). The dominant negative effect of ABCB6/HMT-1 without the NTE domain occurred via the protein interaction with the full-length ABCB6/HMT-1 and not with other ABC transporter-mediated cadmium detoxification pathways. This conclusion was based on the appearance of *hmt-1*-specific refractive inclusions in intestinal cells of Cd-grown wild-type worms expressing ABCB6/HMT-1 without the NTE domain (**Figure 7**). The latter phenotype is specific to Cd-grown *hmt-1* mutant worms and is not manifested by other Cd-sensitive *C. elegans* mutants (Broeks et al., 1996; Vatamaniuk et al., 2001, 2005). In addition, results from yeast-two-hybrid assays showed that ABCB6/HMT-1 without the NTE domain is capable to interact with the full-length ABCB6/HMT-1 (**Figure 5D**). The latter finding is consistent with our previous observations that the NTE domain is not sufficient for the protein–protein interactions of ABCB6/HMT-1 (Kim et al., 2010). To identify additional motif(s) that might be involved in its protein–protein interaction, we tested the function of the C-terminal end, in particular, 43 amino acid region beyond the Walker A ATPase motif, because this part was shown essential for localization of human ABCC1/MRP1 (Westlake et al., 2005). We found that ABCB6/HMT-1^Δ43^ does not interact with the full-length ABCB6/HMT-1 in yeast-two-hybrid assays, does not exert a negative-dominant effect on the full-length polypeptide, and is required for the ability of ABCB6/HMT-1 to rescue the Cd sensitivity of *hmt-1* mutant worms (**Figures 8C,D**). Taken together, the results of structure-function studies suggested that ABCB6/HMT-1/HMT-1 possess interaction motifs within the NTE and the C-terminal 43 amino acids and that both NTE and C-terminal must be present to allow the protein to interact with itself and confer Cd resistance.

## Conclusion

In conclusion, we substantiated our past findings that ABCB6/HMT-1interacts with itself in its native organisms and, at a minimum, homo-dimerized. Further, we showed that ABCB6/HMT-1 resides at the endosomal recycling system and that the NTE domain is essential for routing the protein to the correct endomembrane. We also found that C-terminus is required for ABCB6/HMT-1 interaction with itself. Lastly, both homo-dimerization and correct localization of HMT-1 is required for Cd-detoxification function.

## Author Contributions

SK, AS, and OV designed and carried out the experiments, analyzed the data, and wrote the manuscript. All authors gave final approval for publication.

## Conflict of Interest Statement

The authors declare that the research was conducted in the absence of any commercial or financial relationships that could be construed as a potential conflict of interest.
