# Supplementary material for: N-Terminal Extension and C-Terminal Domains Are Required for ABCB6/HMT-1 Protein Interactions, Function in Cadmium Detoxification, and Localization to the Endosomal-Recycling System in Caenorhabditis elegans
Source: Front Physiol. 2018 Jul 30;9:885. doi: 10.3389/fphys.2018.00885 (PMC6077975; doi:10.3389/fphys.2018.00885)
Supplement: Supplementary file 1 [file Data_Sheet_1.PDF]

Supplementary Material

N-terminal Extension and C-terminal Domains are Required for ABCB6/HMT-1 Protein Interactions, Function in Cadmium Detoxification and Localization to the Endosomal-Recycling System in *Caenorhabditis elegans*

Sungjin Kim[1,3¶], Anuj Kumar Sharma[1,4¶], Olena K. Vatamaniuk[1,2*]

[1]School of Integrative Plant Science, Section of Soil and Crop Sciences, Cornell University, Ithaca, New York, 14853

[2]School of Integrative Plant Science, Section of Plant Biology, Cornell University, Ithaca, New York, 14853

[3]Current Address: Zilkha Neurogenetic Institute, University of Southern California, Keck School of Medicine, Los Angeles, California, 90033

[4]Current Address: Department of Physics, Princeton University, Princeton, New Jersey, 08544

[¶]These authors contributed equally to this work.

*** Correspondence:**
Olena K. Vatamaniuk
okv@cornell.edu

Supplementary Figure 1

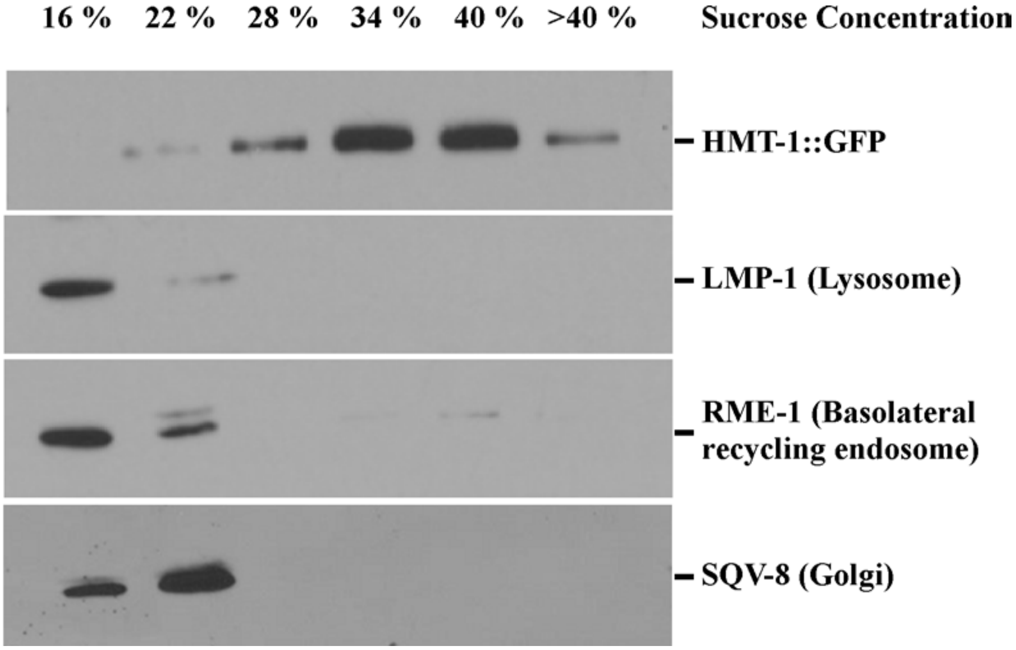

Supplementary Figure 1. Western blot analysis of subcellular localization of ABC6/HMT-1::GFP. Total membrane proteins, isolated from *hmt-1* mutants expressing ABCB6/HMT-1::GFP were fractioned using the sucrose density gradient centrifugation and subjected to western blot analysis. ABCB6/HMT-1::GFP does not co-fraction with the marker for lysosomes or basolateral recycling endosomes, or Golgi apparatus.

Supplementary Figure 2

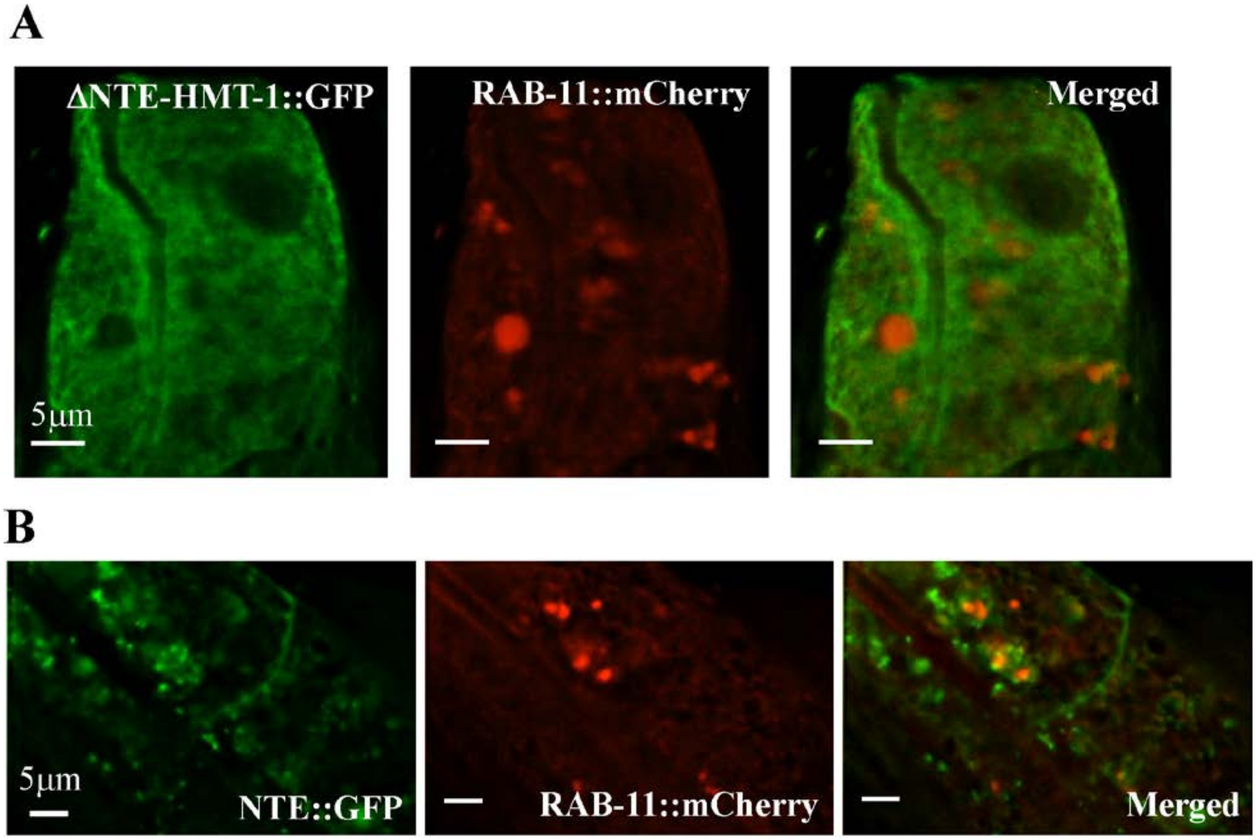

Supplementary Figure 2. Neither ABCB6/HMT-1 lacking NTE nor NTE alone localize to apical recycling endosomes. A. Representative fluorescence microphotograph of *hmt-1* mutant worms co-expressing ΔNTE-HMT-1::GFP and RAB-11::mCherry. **B.** Representative fluorescence microphotograph of *hmt-1* mutant worms co-expressing NTE::GFP construct and RAB-11::mCherry.

Supplementary Figure 3

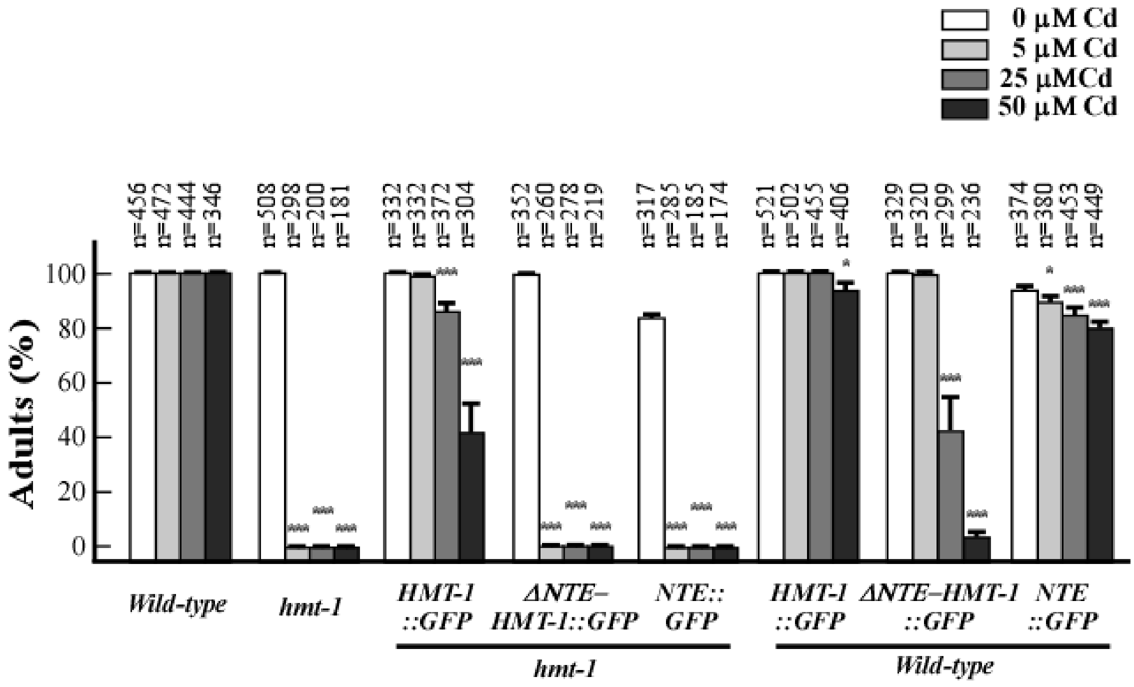

Supplementary Figure 3. ΔNTE-HMT-1 exerts a dominant negative effect on the ability of endogenous HMT-1 to rescue Cd sensitivity of *hmt-1* mutant worms. The percentages of wild-type worms or *hmt-1* mutant expressing the indicated constructs that had reached the adult stage when grown in the presence of the indicated concentrations of cadmium (Cd). The total number of worms tested (n) is shown above each bar. The error bar shows S.D. Asterisks indicate statistically significant differences (*$p < 0.05$, **$p < 0.01$).

Supplementary Figure 4

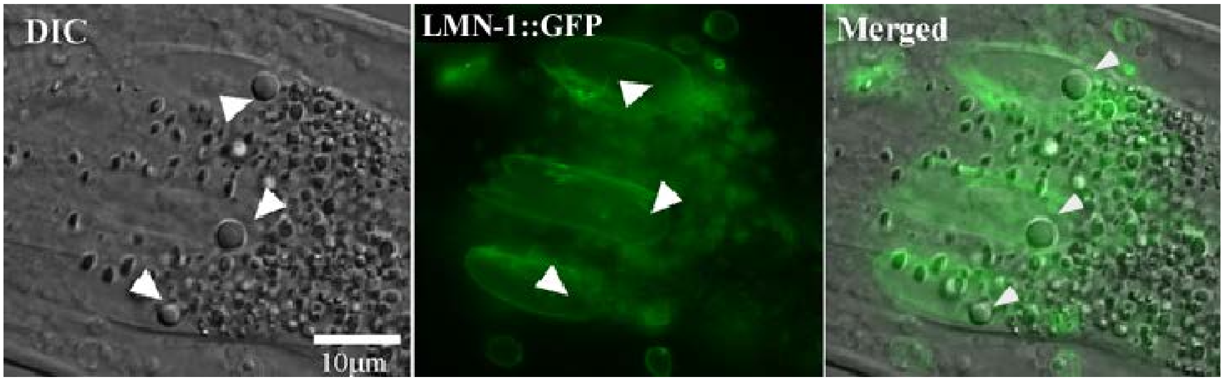

Supplementary Figure 4. The refractile inclusion generated in Cd-grown *hmt-1* mutant worms co-localize with the nucleus. The nuclear envelop marker, LMN-1::GFP, was expressed in the *hmt-1* mutant and transgenic worms were grown on solid NGM medium supplemented with 50 µM $CdCl_2$. White arrowheads indicate the refractile inclusions in intestinal cells of hmt1;LMN-1::GFP worms. We note that these inclusions are formed in the presence of Cd in worms lacking functional HMT-1 but not in other Cd-sensitive mutants of *C. elegans.* A middle panel shows the localization of GFP-mediated fluorescence resulting from the expression of the nuclear envelop marker, LMN-1::GFP in *hmt-1* mutants.
